# Supplementary material for: Tipping the balance: A systematic review and meta-ethnography to unfold the complexity of surgical antimicrobial prescribing behavior in hospital settings
Source: PLoS One. 2022 Jul 20;17(7):e0271454. doi: 10.1371/journal.pone.0271454 (PMC9299309; doi:10.1371/journal.pone.0271454)
Supplement: S1 File — (DOCX) [file pone.0271454.s002.docx]

**Full literature search strategies**

**Database: MEDLINE and MEDLINE-in-Process**

Host: Ovid

Data Parameters: 1946 to May 29 2020

Date Searched: 1/6/2020

Searcher: SR

Hits: 384

| 1 | ((surgery or surgical or theatre or operat*) adj3 (nurse* or nursing* or doctor* or pharmacist* or trainee* or registrar* or junior* or medic* or physician* or consultant* or team* or group* or profession*)).tw. |
| --- | --- |
| 2 | (surgeon* or anaesthetist* or anesthetist*).tw. |
| 3 | exp Surgeons/ |
| 4 | exp Anesthetists/ |
| 5 | Perioperative Nursing/ |
| 6 | Pharmacists/ |
| 7 | or/1-6 |
| 8 | (prescrib* or prescrip* or "decision making" or administer or administration).tw. |
| 9 | ((drug* or medicine* or medication*) adj2 (administ* or utili?ation or error* or use*)).tw. |
| 10 | exp Prescriptions/ |
| 11 | exp Drug Utilization/ |
| 12 | Inappropriate Prescribing/ |
| 13 | Prescription Drugs/ |
| 14 | Drug Prescriptions/ |
| 15 | Medication Errors/ |
| 16 | exp Decision Making/ |
| 17 | or/8-16 |
| 18 | (antimicrobial* or "anti microbial*" or antibiotic* or "anti biotic*" or antibacterial* or "anti bacterial*" or antiviral* or "anti viral*" or antifungal* or "anti fungal*" or "antiinfective*" or "anti infective*" or antiparasitic* or "anti parasitic*").tw. |
| 19 | Anti-Infective Agents/ |
| 20 | exp Anti-Bacterial Agents/ |
| 21 | exp Antifungal Agents/ |
| 22 | exp Anti-Infective Agents, Urinary/ |
| 23 | exp Antiparasitic Agents/ |
| 24 | exp Antiviral Agents/ |
| 25 | Antibiotic Prophylaxis/ |
| 26 | Post-Exposure Prophylaxis/ |
| 27 | Pre-Exposure Prophylaxis/ |
| 28 | or/18-27 |
| 29 | 7 and 17 and 28 |
| 30 | interview:.mp. |
| 31 | experience:.mp. |
| 32 | qualitative:.tw. |
| 33 | 30 or 31 or 32 |
| 34 | 329 and 33 |

**Database: Embase**

Host: Ovid

Data Parameters: 1974 to 2020 May 29

Date Searched: 1/6/2020

Searcher: SR

Hits: 2840

| 1 | ((surgery or surgical or theatre or operat*) adj3 (nurse* or nursing* or doctor* or pharmacist* or trainee* or registrar* or junior* or medic* or physician* or consultant* or team* or group* or profession*)).tw. |
| --- | --- |
| 2 | (surgeon* or anaesthetist* or anesthetist*).tw. |
| 3 | Perioperative Nursing/ |
| 4 | (prescrib* or prescrip* or decision* or administer or administration).tw. |
| 5 | ((drug* or medicine* or medication*) adj2 (administ* or utili?ation or error* or use*)).tw. |
| 6 | (antimicrobial* or "anti microbial*" or antibiotic* or "anti biotic*" or antibacterial* or "anti bacterial*" or antiviral* or "anti viral*" or antifungal* or "anti fungal*" or "antiinfective*" or "anti infective*" or antiparasitic* or "anti parasitic*").tw. |
| 7 | exp surgeon/ |
| 8 | exp anesthesist/ |
| 9 | exp pharmacist/ |
| 10 | 1 or 2 or 3 or 7 or 8 or 9 |
| 11 | prescription/ |
| 12 | drug utilization/ |
| 13 | exp inappropriate prescribing/ |
| 14 | prescription drug/ |
| 15 | exp medication error/ |
| 16 | exp decision making/ |
| 17 | 4 or 5 or 11 or 12 or 13 or 14 or 15 or 16 |
| 18 | exp antiinfective agent/ |
| 19 | exp antifungal agent/ |
| 20 | exp urinary tract antiinfective agent/ |
| 21 | exp antiparasitic agent/ |
| 22 | exp antivirus agent/ |
| 23 | antibiotic prophylaxis/ |
| 24 | post exposure prophylaxis/ |
| 25 | pre-exposure prophylaxis/ |
| 26 | 6 or 18 or 19 or 20 or 21 or 22 or 23 or 24 or 25 |
| 27 | 10 and 17 and 26 |
| 28 | interview:.tw. |
| 29 | exp health care organization/ |
| 30 | experiences.tw. |
| 31 | 28 or 29 or 30 |
| 32 | 27 and 31 |

**Database: APA PsycInfo**

Host: Ovid

Data Parameters: 1806 to May week 4 2020

Date Searched: 1/6/2020

Searcher: SR

Hits: 30

| 1 | ((surgery or surgical or theatre or operat*) adj3 (nurse* or nursing* or doctor* or pharmacist* or trainee* or registrar* or junior* or medic* or physician* or consultant* or team* or group* or profession*)).tw. |
| --- | --- |
| 2 | (surgeon* or anaesthetist* or anesthetist*).tw. |
| 3 | (prescrib* or prescrip* or decision* or administer or administration).tw. |
| 4 | ((drug* or medicine* or medication*) adj2 (administ* or utili?ation or error* or use*)).tw. |
| 5 | (antimicrobial* or "anti microbial*" or antibiotic* or "anti biotic*" or antibacterial* or "anti bacterial*" or antiviral* or "anti viral*" or antifungal* or "anti fungal*" or "antiinfective*" or "anti infective*" or antiparasitic* or "anti parasitic*").tw. |
| 6 | interview:.mp. |
| 7 | experience:.mp. |
| 8 | qualitative:.tw. |
| 9 | 6 or 7 or 8 |
| 10 | surgeons/ |
| 11 | pharmacists/ |
| 12 | 1 or 2 or 10 or 11 |
| 13 | exp "prescribing (drugs)"/ |
| 14 | prescription drugs/ |
| 15 | exp decision making/ |
| 16 | 3 or 4 or 13 or 14 or 15 |
| 17 | exp Antibiotics/ |
| 18 | exp antiviral drugs/ |
| 19 | 5 or 17 or 18 |
| 20 | 12 and 16 and 19 |

**Database: AMED**

Host: Ebsco

Data Parameters: NA

Date Searched: 1/6/2020

Searcher: SR

Hits: 7

| S27 | S5 AND S14 AND S26 |
| --- | --- |
| S26 | S15 OR S16 OR S17 OR S18 OR S19 OR S20 OR S21 OR S22 OR S23 OR S24 OR S25 |
| S25 | SU prophylaxis |
| S24 | SU antiviral |
| S23 | SU antiparasitic |
| S22 | SU anti-infective |
| S21 | SU anti infective |
| S20 | SU antibacterial |
| S19 | SU antifungal |
| S18 | SU antibiotics |
| S17 | SU anti bacterial agents |
| S16 | SU anti infective agents |
| S15 | TX (antimicrobial* or "anti microbial*" or antibiotic^ or *anti biotic*" or antibacterial* or "anti bacterial*" or antiviral* or "anti viral*" or antifungal* or "anti fungal*" or antiinfective* or "anti infective*" or antiparasitic* or "antiparasitic*") |
| S14 | S6 OR S7 OR S8 OR S9 OR S10 OR S11 OR S12 OR S13 |
| S13 | SU decision making |
| S12 | SU medication errors |
| S11 | SU prescription drugs |
| S10 | SU inappropriate prescribing |
| S9 | SU drug utilization |
| S8 | SU prescriptions |
| S7 | TX ((drug* or medicine* or medication*) N2 (administ* or utilization or utilisation or error* or use*)) |
| S6 | TX (prescrib* or prescrip* or decision* or administer or administration) |
| S5 | S1 OR S2 OR S3 OR S4 |
| S4 | SU pharmacist |
| S3 | SU surgeon |
| S2 | TX (surgeon* or anaesthetist* or anesthetist*) |
| S1 | TX ((surgery or surgical or theatre or operat*) N2 (nurse* or nusing* or doctor* or pharmacist* or trainee* or registrar* or junior* or medic* or physician* or consultant* or team* or group* or profession*)) |

**Database: CINAHL**

Host: Ebsco

Data Parameters: NA

Date Searched: 1/6/2020

Searcher: SR

Hits: 1502

| S31 | S27 AND S30 |
| --- | --- |
| S30 | S28 OR S29 |
| S29 | TX thematic analysis |
| S28 | TX grounded theory |
| S27 | S10 AND S17 AND S26 |
| S26 | S3 OR S18 OR S19 OR S20 OR S21 OR S22 OR S23 OR S24 OR S25 |
| S25 | (MH "Pre-Exposure Prophylaxis") |
| S24 | (MH "Antibiotic Prophylaxis") |
| S23 | (MH "Antiviral Agents+") |
| S22 | (MH "Antiparasitic Agents+") |
| S21 | (MH "Antiinfective Agents, Urinary+") |
| S20 | (MH "Antifungal Agents+") |
| S19 | (MH "Antibiotics+") |
| S18 | (MH "Antiinfective Agents+") |
| S17 | S4 OR S5 OR S11 OR S12 OR S13 OR S14 OR S15 OR S16 |
| S16 | (MH "Decision Making+") |
| S15 | (MH "Medication Errors+") |
| S14 | (MH "Drugs, Prescription") |
| S13 | (MH "Inappropriate Prescribing") |
| S12 | (MH "Drug Utilization+") |
| S11 | (MH "Prescriptions, Drug+") |
| S10 | S1 OR S3 OR S6 OR S7 OR S8 OR S9 |
| S9 | (MH "Pharmacists") |
| S8 | (MH "Medical-Surgical Nursing+") |
| S7 | (MH "Anesthetists+") |
| S6 | (MH "Surgeons") |
| S5 | TX ((drug* or medicine* or medication*) N2 (administ* or utilization or utilisation or error* or use*)) |
| S4 | TX (prescrib* or prescrip* or decision* or administer or administration) |
| S3 | TX (antimicrobial* or "anti microbial*" or antibiotic^ or *anti biotic*" or antibacterial* or "anti bacterial*" or antiviral* or "anti viral*" or antifungal* or "anti fungal*" or antiinfective* or "anti infective*" or antiparasitic* or "antiparasitic*") |
| S2 | TX (surgeon* or anaesthetist* or anesthetist*) |
| S1 | TX ((surgery or surgical or theatre or operat*) N2 (nurse* or nusing* or doctor* or pharmacist* or trainee* or registrar* or junior* or medic* or physician* or consultant* or team* or group* or profession*)) |

**Database: Cochrane**

Host: Wiley

Data Parameters: Issue 6 of 12, June 2020

Date Searched: 2/6/2020

Searcher: SR

Hits: 693

| #1 | ((surgery or surgical or theatre or operat*) near/2 (nurse* or nursing* or doctor* or pharmacist* or trainee* or registrar* or junior* or medic* or physician* or consultant* or team* or group* or profession*)):ti,ab,kw |
| --- | --- |
| #2 | (surgeon* or anaesthetist* or anesthetist*):ti,ab,kw |
| #3 | MeSH descriptor: [Surgeons] explode all trees |
| #4 | MeSH descriptor: [Anesthetists] explode all trees |
| #5 | MeSH descriptor: [Perioperative Nursing] explode all trees |
| #6 | MeSH descriptor: [Pharmacists] explode all trees |
| #7 | #1 or #2 or #3 or #4 or #5 or #6 |
| #8 | (prescrib* or prescrip* or "decision making" or administer or administration):ti,ab,kw |
| #9 | ((drug* or medicine* or medication*) near/2 (administ* or utili?ation or error* or use*)):ti,ab,kw |
| #10 | MeSH descriptor: [Prescriptions] explode all trees |
| #11 | MeSH descriptor: [Drug Utilization] explode all trees |
| #12 | MeSH descriptor: [Inappropriate Prescribing] explode all trees |
| #13 | MeSH descriptor: [Prescription Drugs] explode all trees |
| #14 | MeSH descriptor: [Drug Prescriptions] explode all trees |
| #15 | MeSH descriptor: [Medication Errors] explode all trees |
| #16 | MeSH descriptor: [Decision Making] explode all trees |
| #17 | #8 or #9 or #10 or #11 or #12 or #13 or #14 or #15 or #16 |
| #18 | (antimicrobial* or "anti microbial*" or antibiotic* or "anti biotic*" or antibacterial* or "anti bacterial*" or antiviral* or "anti viral*" or antifungal* or "anti fungal*" or "antiinfective*" or "anti infective*" or antiparasitic* or "anti parasitic*"):ti,ab,kw |
| #19 | MeSH descriptor: [Anti-Infective Agents] explode all trees |
| #20 | MeSH descriptor: [Anti-Bacterial Agents] explode all trees |
| #21 | MeSH descriptor: [Antifungal Agents] explode all trees |
| #22 | MeSH descriptor: [Anti-Infective Agents, Urinary] explode all trees |
| #23 | MeSH descriptor: [Antiparasitic Agents] explode all trees |
| #24 | MeSH descriptor: [Antiviral Agents] explode all trees |
| #25 | MeSH descriptor: [Antibiotic Prophylaxis] explode all trees |
| #26 | MeSH descriptor: [Post-Exposure Prophylaxis] explode all trees |
| #27 | MeSH descriptor: [Pre-Exposure Prophylaxis] explode all trees |
| #28 | #18 or #19 or #20 or #21 or #22 or #23 or #24 or #25 or #26 or #27 |
| #29 | #7 and #17 and #28 |

**Database: Web of Science**

Host: Clarivate Analystics

Data Parameters: SCI-Expanded 1900-present; SSCI 1956-present

Date Searched: 2/6/2020

Searcher: SR

Hits: 2/6

| # 10 | #9  AND  #8 |
| --- | --- |
| # 9 | TS=(interview* or  experience*  or  qualitative*) |
| # 8 | #7  AND  #6  AND  #5 |
| # 7 | #4  OR  #3 |
| # 6 | #2  OR  #1 |
| # 5 | TS=(antimicrobial* or  "anti  microbial*"  or  antibiotic*  or  "anti  biotic*"  or  antibacterial*  or  "anti  bacterial*"  or  antiviral*  or  "anti  viral*"  or  antifungal*  or  "anti  fungal*"  or  "antiinfective*"  or  "anti  infective*"  or  antiparasitic*  or  "anti  parasitic*") |
| # 4 | TS=((drug* or  medicine*  or  medication*)  near/2  (administ* or utili?ation or error* or use*) ) |
| # 3 | TS=(prescrib* or  prescrip*  or  "decision  making"  or  administer  or  administration) |
| # 2 | TS=(surgeon* or  anaesthetist*  or  anesthetist*) |
| # 1 | TS=((surgery or  surgical  or  theatre  or  operat*)  near/2  (nurse* or nursing* or doctor* or pharmacist* or trainee* or registrar* or junior* or medic* or physician* or consultant* or team* or group* or profession*) ) |
